# Prevalence of Autism Spectrum Disorder in the Centro region of Portugal: a population based study of school age children within the ASDEU project

**DOI:** 10.3389/fpsyt.2023.1148184

**Published:** 2023-08-30

**Authors:** Célia Rasga, João Xavier Santos, Cátia Café, Alexandra Oliveira, Frederico Duque, Manuel Posada, Ana Nunes, Guiomar Oliveira, Astrid Moura Vicente

**Affiliations:** ^1^Instituto Nacional de Saúde Doutor Ricardo Jorge, Lisboa, Portugal; ^2^Faculty of Sciences, University of Lisboa, BioISI - Biosystems & Integrative Sciences Institute, Lisboa, Portugal; ^3^Unidade de Neurodesenvolvimento e Autismo, Serviço do Centro de Desenvolvimento da Criança, Centro de Investigação e Formação Clínica, Hospital Pediátrico, Centro Hospitalar e Universitário de Coimbra, Coimbra, Portugal; ^4^Faculty of Medicine, University Clinic of Pediatrics and Coimbra Institute for Biomedical Imaging and Translational Research, University of Coimbra, Coimbra, Portugal; ^5^Institute of Rare Diseases Research, IIER, Instituto de Salud Carlos III, ISCIII, Madrid, Spain; ^6^Departamento de Física, Faculdade de Ciências, Universidade de Lisboa, Lisboa, Portugal

**Keywords:** ASDEU study, autism, epidemiology, population survey, prevalence

## Abstract

**Introduction:**

Accurate prevalence estimates for Autism Spectrum Disorder (ASD) are fundamental to adequately program medical and educational resources for children. However, estimates vary globally and across Europe, and it is therefore wise to conduct epidemiological studies in defined geo-cultural contexts.

**Methods:**

We used a population screening approach to estimate the prevalence of ASD in the Centro region of Portugal, using a harmonized protocol as part of the Autism Spectrum Disorders in the European Union (ASDEU) project.

**Results:**

The overall prevalence was estimated at 0.5% (95% CI 0.3–0.7), higher in schools with Autism Units (3.3%, 95%CI 2.7–3.9) than in regular schools (0.3%, 95% CI 0.1–0.5) or schools with Multiple Disability Units (0.3%, 95% CI 0.04–0.6).

**Discussion:**

The results indicate that the diagnosis of ASD is followed by the most effective educational policies in Centro Region. The variability in prevalence estimates across the different regions from the ASDEU project, and globally, is discussed.

## 1. Introduction

Autism Spectrum Disorder (ASD) is a neurodevelopmental disorder defined by social and communication impairments, and rigid and repetitive patterns of behavior and interests (1, 2) that emerge very early in life. The clinical manifestations of ASD can vary broadly with age, cognitive ability and co-morbidities, and the recognition of this wide variability led to the concept of an autism spectrum. The etiology of ASD and the origin of this clinical heterogeneity are still poorly understood. Heritability estimates support an important role of genetic factors (3). However, less than 25% of ASD cases carry an identifiable etiological genetic alteration, and most patients remain idiopathic (4). Evidence for an impact of other factors, notably epigenetic mechanisms (5) and environmental exposure (6, 7), has been accumulating over time, contributing to the perceived complex etiological architecture of ASD.

The first prevalence studies of ASD were carried out between 1960 and 1980, when a strict definition of autism as a very severe condition, commonly associated with intellectual disability, was accepted. These earlier studies reported a prevalence of approximately 4 to 5 cases per 10,000 children (8, 9). Population-based studies carried out since the mid-1990s, however, consistently show a higher prevalence of ASD. In 2022, a systematic review reported a global prevalence of 1% (10). However, prevalence estimates vary hugely among different countries, from very high values in South Korea (2.64%) (11) and in the United States of America (2.50%) (12, 13) to lower estimates in the Haarlem (0.84%) and Utrecht (0.57%) regions from the Netherlands (14), in Germany (0.38%) (15), and in the Kolkata region in India (0.23%) (16).

The reasons underlying the prevalence increase observed over time and the regional variability of ASD prevalence are still unclear. In part, the apparent increase in ASD prevalence may be related to the broadening of diagnostic concepts resulting from changes in diagnostic criteria in the various versions of the Diagnostic and Statistical Manual of Mental Disorders (17). However, studies that directly quantify these effects are not available. Greater awareness in society, parents and professionals may also contribute to the increasing rates of ASD (18). On the other hand, some authors argue that the increase is real and may be attributable to factors that have evolved in recent years, such as early life exposure to some environmental toxicants or advanced parental age (19, 20).

Heterogeneous study designs, including studies based on registries or based on community surveys, can lead to differences in prevalence estimations, over time and regionally (21–23). In registry studies, the absence of standardized methodology to estimate ASD prevalence means that each study may reflect local educational and health services infrastructures, or social policies for children with disabilities. Improved documentation procedures or evolving diagnostic criteria challenge registry designs (24). On the other hand, in community surveys in schools, participation rates from teachers and parents will affect results. For instance, parents of children with ASD tend to participate more than non-ASD parents, leading to lower ASD rates in nonparticipants than in participants (25). Case definitions may not be uniform, as studies rely on different diagnostic tools: electronic records, autism special education eligibility, questionnaires filled by caregivers or individual clinical assessments. This has an impact on the quality of the collected data, on the validity of the diagnoses, especially by unexperienced clinicians, and on the screening and diagnostic confirmation tools, which should rely on independent procedures (25). Overall, heterogeneous study designs, variable diagnostic protocols, a fluctuating awareness of the disorder and cross-cultural differences, have made a consensual prevalence estimate for ASD difficult to achieve so far. Nevertheless, epidemiological studies are still essential for the implementation of adequate health and education management strategies in ASD.

In this study we adopted a protocol that is part of the wider Autism Spectrum Disorders in European Union (ASDEU) project. The ASDEU project was carried out by a consortium of 14 European countries and aimed to further our understanding regarding the burden of ASD in Europe, as well as the common practices for its diagnosis and management. One of its main objectives was to estimate the prevalence of ASD, using harmonized methodologies, in specific regions from 12 participating countries. A common methodology for prevalence estimation through community surveys was applied to specific regions in 8 countries: Austria, Bulgaria, Ireland, Italy, Poland, Portugal, Romania and Spain. In all regions the target population were children aged 7–9 years, and screening was carried out in schools. Diagnosis was performed through direct clinical assessment by neurodevelopmental pediatricians. Also, within ASDEU, 4 additional countries, namely Denmark, Finland, France and Iceland, used population health registries for prevalence estimation.

The ASD prevalence in the ASDEU countries that used national or regional registries were estimated at 1.26% in Denmark, 0.77% in Finland and 3.13% in Iceland, as well as 0.48% in South-East France and 0.73% in South-West France (26). In the Tuscany region of Italy, and in the Basque Country in Spain, the prevalence was determined by surveying children in primary schools following the ASDEU community protocol, and prevalence estimated at 1 and 0.59%, respectively (27, 28).

In the present study, also performed in the context of the ASDEU project, we carried out a cross-sectional epidemiological study to determine the prevalence of ASD in the Centro region from Portugal. The study followed the ASDEU methodology for prevalence estimation through community survey, in which children with ASD were identified by screening primary schools using a nomination procedure by teachers, followed by diagnosis through direct observation by an expert clinical team in autism and other neurodevelopmental disorders (29). All Portuguese children aged 7 to 9 years are registered in primary schools, where most teachers follow the same class for up to 4 years (the span of the first cycle of education), and therefore know their pupils very well. Generally, these teachers also have the competences and/or experience to detect potential neurodevelopmental problems. Moreover, specialized support at public schools is offered to children, with assistance by special education teams, adapted education programs or by enrolling in schools that have special education units specifically suited to their needs. This was therefore a sensible screening strategy.

A previous nation-wide epidemiological study for ASD in mainland Portugal and the Azores, which included the Centro region, was carried out using a very similar study design in 2001, by this same research team. The national ASD prevalence estimated in 2001 was 0.92% (95% CI 0.81–1.0) (30). The present study allows the comparison of ASD prevalence at two time points, with a time gap of approximately 17 years. To be able to draw conclusions regarding the status and evolution of ASD in Portugal, we further carried out a detailed demographic and clinical characterization of the screened population, to gather information of relevance for planning educational and health resources for individuals with ASD.

## 2. Methods

### 2.1. Target population

The target population consisted of children of both sexes and any ethnicity, living in a well-defined geographic region of Portugal, the Centro region, born between the 1^st^ of January, 2007 and the 31^st^ of December, 2009 (thus aged between 7 and 9 years in 2017) attending primary school in the 2016-2017 school year.

Portugal currently has a population of nearly 10.3 million inhabitants. The country is divided into seven major regions, corresponding to subdivisions defined by the Nomenclature of Territorial Units for Statistics, the European Union geocoding standard (NUTS-II, *Nomenclatura das Unidades Territoriais para Fins Estatísticos*)^1^. The Centro region, located in mainland Portugal, was selected for this study for several reasons: (1) the Centro region spans an area of 28,200 km^2^, covering 31.3% of mainland Portugal, and has a population of 2.2 million inhabitants, representing 21.5% of the total mainland population^2^; (2) this region is typical of the Portuguese demography, social background and geo-social diversity, with both rural and urban settings, and coastal and interior regions; (3) the Pediatric Hospital – Centro Hospitalar e Universitário in Coimbra, partner in the ASDEU project, is part of the university teaching hospital and is the reference pediatric hospital in Centro region, thus providing assistance and holding the clinical registers of a large percentage of the children with ASD that reside in this region; the clinical team that works with ASD in this hospital is highly experienced in diagnosing neurodevelopmental disorders and a reference for autism in Portugal for more than two decades; (4) other hospitals in Centro region that support children with neurodevelopmental problems work in close contact with this reference hospital; (5) the clinical team has strong ties with schools in the country, specially in Centro region, and with the special education teams that support children with ASD.

All legal representatives of the participants signed an informed consent to participate in the study and approval for the study was obtained from the Ethical Commission for Health at the National Institute for Health Doutor Ricardo Jorge.

### 2.2. School selection

In Portugal, primary education is mandatory, and free for all children in the network of public schools. Private schools are also available. Special education policies prioritize integration in mainstream schools, and across Portugal there is a network of educational support services in public primary schools, available for children with special education needs. Most schools in the public network that enroll children with neurodevelopmental disabilities have units for children with special education needs, namely “Units of structured education for support and inclusion of students with ASD” (from here on designated as Autism Units), or “Units of specialized support for education of students with Multiple Disability and congenital blindness and deafness” (from here on designated as Multiple Disability Units). These units congregate the human and material resources necessary to provide the best support to these students. The support services include teams for coordination of educational support, special education teachers and psychological support services.

Aiming at a wide-ranging screening for children with ASD, we targeted three school typologies: (1) regular primary schools, public and private, without special needs units (hereby designated regular schools); (2) primary schools with Autism Units; (3) primary schools with Multiple Disability Units. The Ministry of Education, through *Direção Geral de Estabelecimentos Escolares* (DGEE), provided the inventory of primary schools and the list of schools with Autism and Multiple Disability Units in Centro region for the school year of 2016–2017. According to DGEE, in this school year, there were a total of 1,129 primary schools in the Centro region of Portugal, of which 1,054 were regular primary schools and 75 were schools with Autism Units (*N* = 41) or Multiple Disability Units (*N* = 34). These 1,129 schools were attended by 77,717 students from the first cycle of education, which spans 4 years and includes children aged 6–10 years. This included 5,180 and 4,952 children attending the 75 schools with Autism or Multiple Disability Units in the region, respectively. Given the large number of primary schools in the Centro region, we opted to screen all schools with Autism or Multiple Disability Units, but to randomly select approximately 10% of the regular schools without special needs units, targeting a population of at least 10,000 screened children. This random selection was carried out using weighted random sampling without replacement, through R software environment.

The selection of regular primary schools resulted in 122 schools, distributed by district and geographic region. All 75 schools with special education units were contacted. Overall, a total of 197 schools were selected for screening. The exact number of students aged 7 to 9 attending each screened school in 2016–2017 was provided during the screening procedure, as each teacher was asked to specify the total number of children attending their class in that age range.

Authorization to contact the schools was obtained from the Ministry of Education. Because schools are administratively and geographically organized in school clusters, we initially sought permission from the school cluster administrations to contact each selected school. Once permission was obtained, all schools received a package by regular mail containing several copies of the documentation, which included a letter to the school director, an information note to teachers, a consent form for teachers to participate in the study and the Teacher Nomination Form. We followed up with emails and phone contacts, as well as on-site visits whenever necessary.

### 2.3. Nomination strategy – The teacher nomination form

To identify children with ASD in Centro region, we used the Teacher Nomination Form (TNF) (29). This form is a questionnaire developed with the objective of identifying children at risk for ASD in a timely, reliable and cost-effective manner. The TNF was previously translated to Portuguese and validated by questioning teachers from schools known to be attended by children with ASD. The TNF takes teachers through six behavioral characteristics associated with ASD, asking them to consider whether any of the children in their classroom displays any of these characteristics and, if so, how many children. The 6 characteristics are expressed as follows: “Socially awkward”; “Does not seem to understand the feelings of others”; “Talks a lot about own interests, but not very good at conversations.”; “Does not really chat to be friendly.”; “Not very flexible - Tends to insist on certain rules and routines.”; and “Is intensely interested in just a few topics or activities.”

Teachers were asked to nominate no more than four children from their classroom that showed one or more of the behavioral characteristics specified in the TNF and rank them in descending order regarding the number of displayed behaviors. In order not to miss milder cases, even when the teacher considered that none of the children in the classroom showed any of these behaviors, they could always nominate at least one child, marking which of the six characteristics was at least slightly shown, and the one that best described the single nominated child. However, nomination was not mandatory. For each nominated child, teachers were also asked about other relevant behavioral difficulties, any special educational needs and ongoing educational support (28, 29).

At this stage none of the children nominated by teachers were identified by name. Teachers were asked to attribute a code to each child and request consent from the parents or legal representatives for further participation, by handing out an information leaflet and a consent form. Any questions from the parents were answered by teachers, who could contact the research team to clarify any issues. After obtaining parental consent, the teachers disclosed the identities of the children to the clinical team, who contacted the families for assessment.

### 2.4. Clinical evaluation and diagnosis

All children nominated by teachers and with consent to participate in the study were evaluated at the Pediatric Hospital in Coimbra, the reference hospital for autism in the Centro region. Formal assessment included the Autism Diagnostic Observation Schedule-Generic (ADOS-G) (31) and/or the Autism Diagnostic Interview – Revised and DSM-5 criteria (1). Intellectual/developmental characterization was performed using the Griffiths Mental Scale–II (32) for children with a cognitive age below their chronological age, or the Portuguese version of the Wechsler Intelligence Scale for Children (WISC) (33). Adaptive behavior was assessed using the Vineland Adaptive Behavior Scale (VABS) (34).

A fraction of the children was already routinely followed by the clinical team, so their clinical information was obtained from the hospital records. Whenever necessary to complete the protocol, an assessment was scheduled for these children, or they were assessed at their next scheduled visit to the hospital.

The information on participants was introduced in a database and regularly updated. Participants were given a code, so that identifying information did not figure in this database. A file with the code key was separately stored. Data quality control was performed through regular validations by three research team members.

### 2.5. Prevalence estimation

Prevalence estimation was carried out separately for three populations according to school typology: regular schools, schools with Autism Units and schools with Multiple Disability Units.

For the estimation of the prevalence in each of these sets the following formula was used:


$$P=\frac{a}{n}\frac{d}{e}$$

where *n*, *a*, *e,* and *d* stand, respectively, for the numbers of sampled, nominated, evaluated and diagnosed children in each school type.

For sample variance (*s*^2^) and population variance (*σ*^2^) the following formulas were used:


$$s^{2}=\frac{d\left(1-\frac{d}{e}\right)}{e-1}$$

$$\sigma^{2}=\frac{N-n}{N}\frac{1}{n}{\left(\frac{d}{e}\right)}^{2}\frac{a}{n}\left(1-\frac{a}{n}\right)+\left(\frac{a-1}{n}-\frac{e-1}{N}\right)\left(\frac{a}{n}\right)\left(\frac{s^{2}}{e}\right)$$

in which *N* represents the total population enrolled in each type of school. The 95% CI is then given by *P ± zσ*, where *z* ≅ 1.96 represents the z-score for 95%.

We further estimated a final prevalence value (*Pt*), for the whole targeted population, using the following formula:


$$Pt=\frac{\Sigma_{i}\left(N_{i}P_{i}\right)}{Nt}$$

in which the sum goes over the three strata, *N_i_* represents the total population in each school typology (*N1, N2, N3*), *P*_i_ the prevalence calculated for each school typology (*P1, P2, P3*) and *Nt* stands for the total population targeted.

Population variance was determined as follows:


$$\sigma_{t}{}^{2}=\frac{\Sigma_{i}{\left(N_{i}\sigma_{i}\right)}^{2}}{Nt^{2}}$$

in which the sum goes over the three strata and $\;\sigma_{i}{}^{2}$ is the population variance calculated for each school type. As before, the 95% CI for the total prevalence is then given by *Pt* $\pm z\;\sigma_{t}$ .

**Figure 1:**
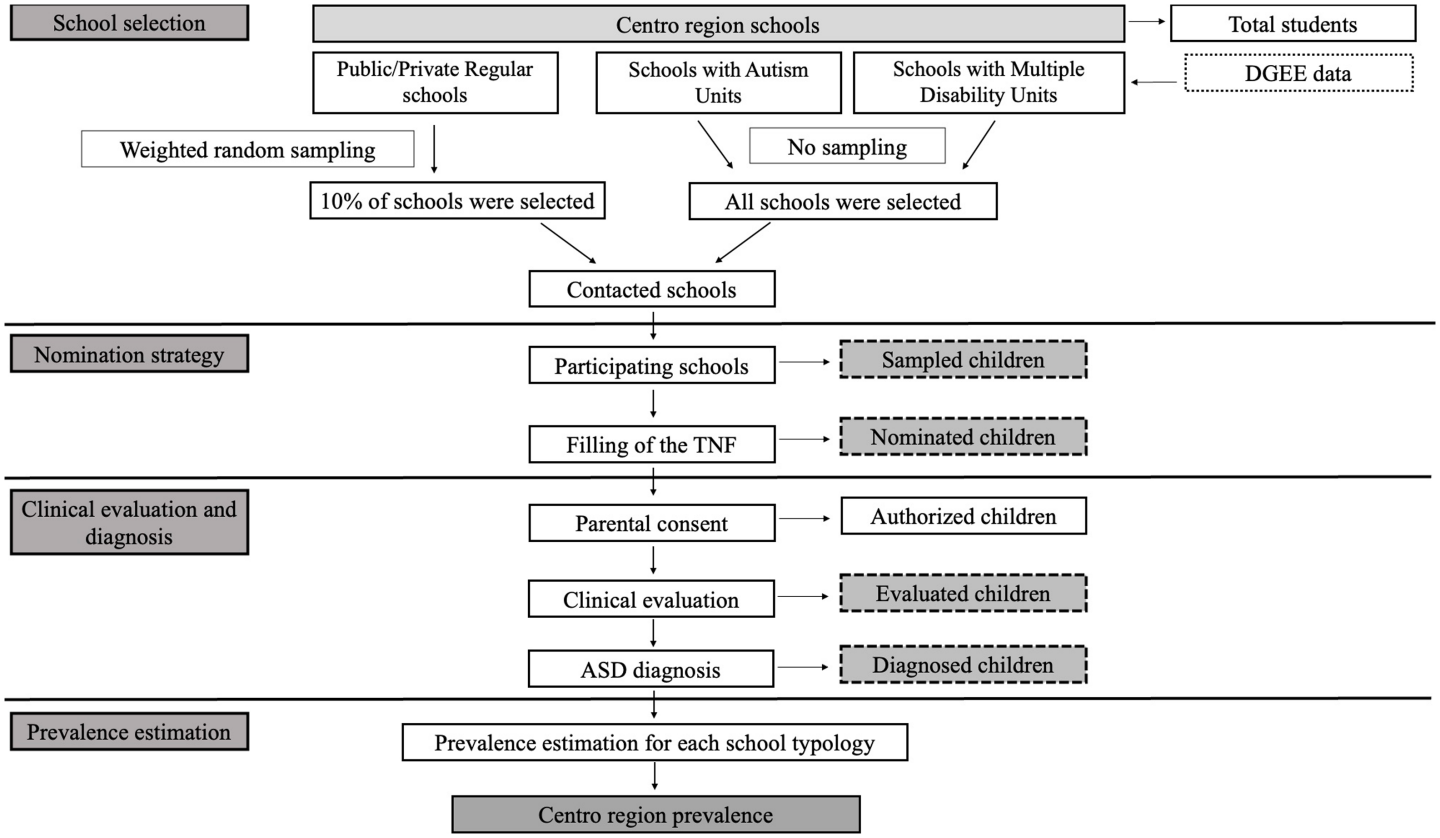
Flowchart summarizing the strategy used to screen Centro region for the estimation of ASD prevalence in the school year of 2016–2017. ASD, Autism Spectrum Disorder; DGEE, Direção Geral de Estabelecimentos Escolares.

The strategy used to screen Centro Region is shown in Figure 1. All analyses were performed using the R software environment.

## 3. Results

### 3.1. Screening phase

**Figure 2:**
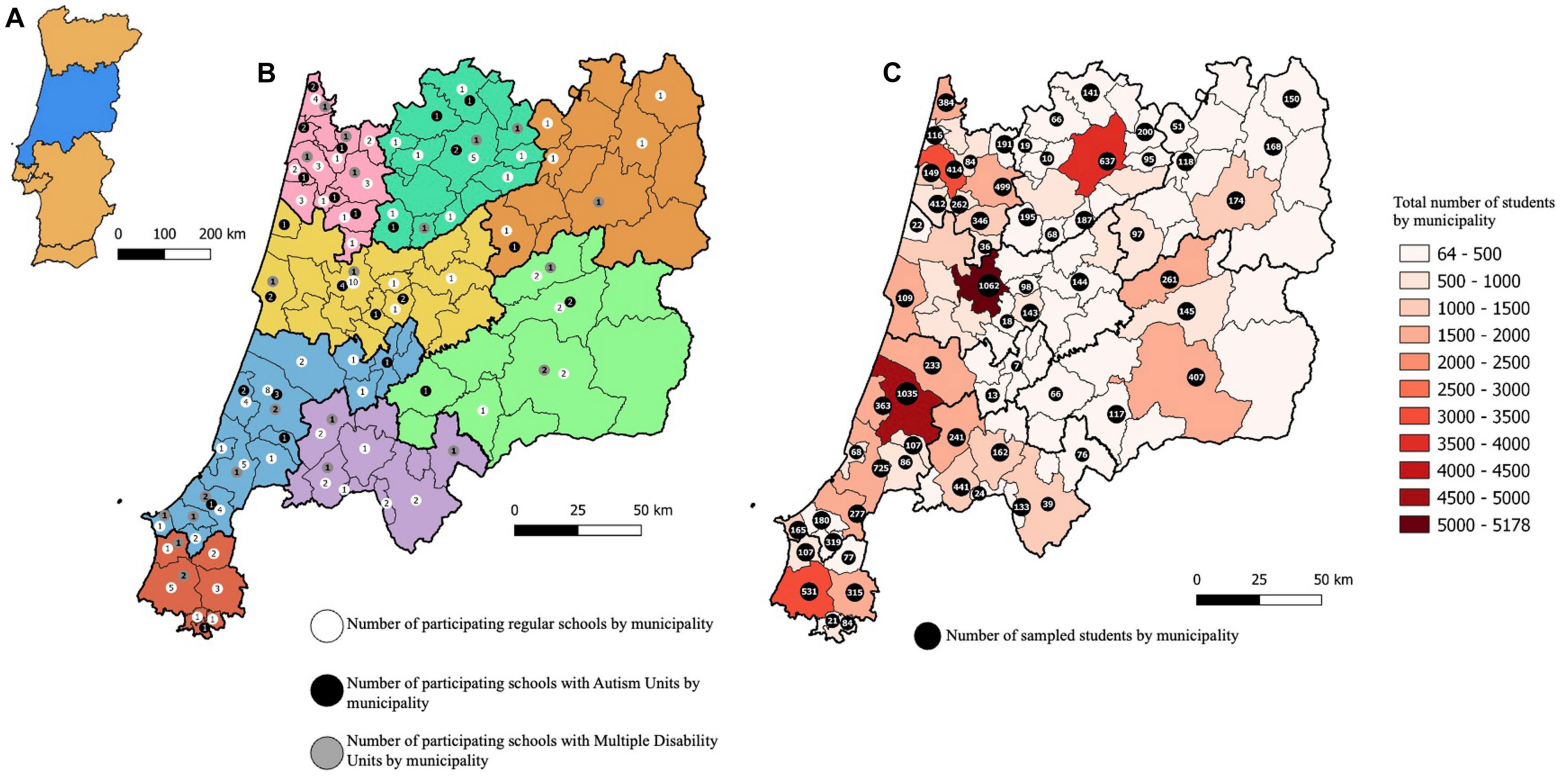
Distribution of participating schools and sampled students along Centro region. **(A)** Geographical location of Centro region (in blue) in mainland Portugal. **(B)** Distribution of participating schools, by typology, by municipality along Centro region. **(C)** Total numbers of 7–9 years old children enrolled in all schools by municipality (color gradient) and numbers of sampled children by municipality (black dots).

Out of 197 selected schools, 173 agreed to participate in the study, yielding an overall high participation rate of 88% (Table 1). This high rate of participation resulted from extensive efforts to repeatedly contact non-responding schools, by email and telephone, followed by *on-site* visits. An adequate timing for the study was also crucial, as school visits were programmed for the end of the school year so that teachers were no longer overwhelmed with classes, evaluations, and other academic activities. The participation rate was higher for regular schools (91%) and schools with Autism Units (88%), and lower for schools with Multiple Disability Units (76%) (Table 1). As expected, the participating schools were more concentrated in coastal municipalities when compared to interior municipalities, thus being representative of the real population distribution along the surveyed region (Figures 2A,B).

**Table 1:** Numbers of participating and selected schools and numbers of children screened per type of school.

**School typology**	**Participating (*n*)/selected (*n*) schools (%)**	**Children screened by teachers in participating schools (*n*)**
Regular schools	111/122 (91%)	8,451
Schools with Autism Units	36/41 (88%)	2,425
Schools with Multiple Disability Units	26/34 (76%)	2,814
Total	173/197 (88%)	13,690

A total of 716 teachers in the participating schools filled the TNF relative to 13,690 children, representing approximately 18% of the total population of children in the 7 to 9 age range attending all schools in Centro region. This study sample included 8,451 children in regular primary schools (public and private), and 2,425 and 2,814 children attending schools with Autism or Multiple Disability Units, respectively (Table 1). From these 13,690 children, 195 (1.4%) were nominated by teachers using the TNF: 60 (31%) children attended regular primary schools, while 114 (58%) and 21 (11%) attended schools with Autism or Multiple Disability Units, respectively. Again, the distribution of students along the surveyed region was, as expected, higher in coastal municipalities (Figure 2C).

The rate of parental consent was relatively low, as only 96 (49%) of the parents of nominated children consented to participate: 16 (17%) children from public and private regular schools, 72 (75%) children from schools with Autism Units and 8 (8%) children from schools with Multiple Disability Units. For ethical reasons, parents could not be contacted directly by the research team before consent, so it is not possible to be certain regarding the causes of the lower rate of authorization. Teachers referred non-acceptance by the parents of the child’s condition as the most frequent reason why consent could not be obtained. A preliminary comparison of the number and profile of TNF behavioral characteristics that were selected by teachers for children with and without consent did not show any significant differences (α = 0.05), and neither did the “most relevant item” selected.

### 3.2. Clinical assessment and diagnosis

Following the teacher nomination, the Pediatric Hospital in Coimbra contacted the 96 families who consented to participate for assessment and diagnosis. From these, 11 children did not visit the hospital for assessment (3, 7 and 1 children from regular, Autism Unit and Multiple Disability Unit schools, respectively). Thus, clinical evaluation was performed in 85 (89%) children: 13 (15%) children from regular schools, 65 (77%) children from schools with Autism Units and 7 (8%) children from schools with Multiple Disability Units. Overall, the rate of children evaluated (number of children evaluated/number of children nominated) was 44%. This rate was higher for schools with Autism Units (57%), supporting the trend for greater cooperation from parents of children attending these schools, than for schools with Multiple Disability Units (33%) and regular schools (22%).

Diagnosis of ASD according to DSM-5 criteria was confirmed in 55 (65%) of the assessed children: 6 (11%) children from regular schools, 46 (84%) from schools with Autism Units and 3 (5%) children from schools with Multiple Disability Units. Diagnostic rates were clearly higher in schools with Autism Units (71%) than in regular schools (46%) or schools with Multiple Disability Units (43%). The overall rate of children with confirmed diagnosis (n° with positive diagnosis/n° of children evaluated) was 65%.

**Figure 3:**
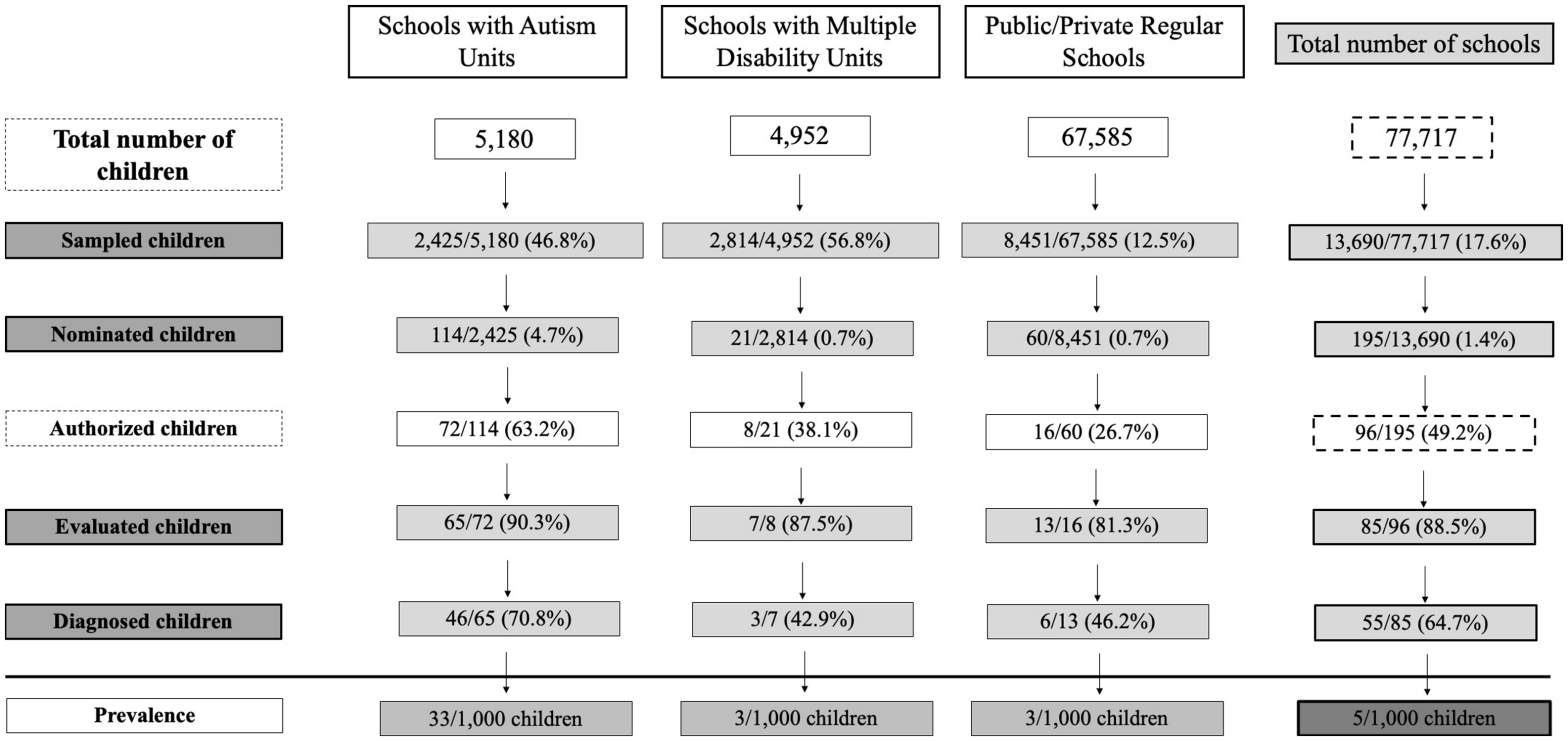
Flowchart summarizing the results from the sampling, nomination, clinical evaluation and prevalence estimation stages.

The results from the sampling, nomination, clinical evaluation and prevalence estimation stages for each school type are summarized in Figure 3.

### 3.3. Prevalence of ASD

Prevalence of ASD was analyzed globally and for each school typology. Table 2 shows the prevalence estimates of ASD in Centro region. The global prevalence was 0.5% (95% CI 0.3–0.7). The prevalence was significantly higher in schools with Autism Units than in the two other school typologies (Table 2), indicating that most of the children with ASD in Centro region were enrolled in the educational programs that will provide them with the best chances of development.

**Table 2:** Estimated prevalence of ASD, global and in the three school settings, in Centro region in the school year 2016–2017, with 95% confidence intervals.

	*N*	*n*	*a*	*e*	*d*	*P* (%)	*σ*^2^	95% CI
Regular schools	67,585	8,451	60	13	6	0.3	1.16×10^−06^	0.1–0.5
Schools with Autism Units	5,180	2,425	114	65	46	3.3	1.01×10^−05^	2.7–3.9
Schools with Multiple Disability Units	4,952	2,814	21	7	3	0.3	2.00×10^−06^	0.04–0.6
Global	77,717	13,690	195	85	55	0.5	9.27×10^−07^	0.3–0.7

*N*, total population; *n*, sampled children; *a*, nominated children; *e*, evaluated children; *d*, diagnosed children; *P*, prevalence; *σ*^2^, population variance.

### 3.4. Clinical characterization of the children diagnosed with ASD

A total of 85 children were formally assessed, and ASD diagnosis was confirmed in 55 children. Forty-one were males and fourteen were females, yielding a male to female ratio of 2.9:1. The median age of diagnosis was 3 years and 9 months. Using ADOS cutoffs, 43 children were classified as having Autism and 9 as having ASD, thus presenting milder symptoms.

Cognitive/developmental characterization was carried out in 46 (83.6%) of the diagnosed children. Half showed a development quotient (DQ) or intelligence quotient (IQ) ≥ 70 and the other half had DQ or IQ <70. Of the 55 children assessed, 38 (69.1%) children were verbal and 17 (30.9%) were non-verbal.

Other co-occurring conditions were documented in 23 (41.8%) children. Twenty children had Intellectual Disability (ID), 2 had epilepsy (1 also included in the ID group) and 2 children had macrocephaly.

Information about the onset of symptoms was gathered for 51 children (92.7%). Parents or caregivers reported developmental problems within the first year of the child’s life in 14 children (27.5%), whereas in 30 patients (58.8%) problems were only apparent in the second year and in 7 (13.7%) children in the third or fourth year of life. There was information about the initial symptoms in 53 children. Language delay was the first symptom in 21 children, developmental delay and regression in 11, impairments in social communication and interaction in 7, behavior problems in 6, language regression (defined by the loss of at least five words that had been used regularly for 3 months) in 5, walking in toes in 2 and dietary restrictions in 1 child.

Regarding family history of neurodevelopmental disorders, the 55 diagnosed children included one pair of twin sisters and one pair of opposite sex siblings. Four other children had siblings with an ASD diagnosis, 2 children had siblings with language delay, 6 children had family history of learning disabilities (5 related to parents and 1 related to a sibling) and 1 child had an uncle with language delay,

From the 85 assessed children, in 30 the diagnosis of ASD was not confirmed. In this group, the male to female ratio was 9:1 (27 males, 3 females). Cognitive/developmental characterization was carried out in 12 of these children, with 5 having DQ or IQ <70. Seven of these 30 children were already patients of the clinical assessment team at the Pediatric Hospital in Coimbra and had been diagnosed with Intellectual Disability (*N* = 3), Attention deficit/hyperactivity disorder (*N* = 1), Selective Mutism (*N* = 1), Communication disorder (*N* = 1) and Oppositional defiant disorder (*N* = 1). For the other children not followed at the Pediatric Hospital, it was not possible to retrieve clinical files with diagnostic information.

## 4. Discussion

The aim of the present study was to estimate the prevalence of ASD in Centro region of Portugal. For this we adopted a multistage strategy, defined in the context of the ASDEU project, based on the screening of a large sample of children aged 7 to 9 years in an educational setting, followed by clinical evaluation of nominated children. We estimated a global prevalence for the region of 0.5%, with a relatively narrow 95% CI of 0.3–0.7 that indicates a valid precision in the estimation.

Prevalence was separately estimated for schools with and without special needs units, allowing us to understand if children with ASD are correctly integrated in the education system. The estimates were 11 times higher in schools with Autism Units (3.3%), when compared to schools without special needs units (0.3%) or to schools with Multiple Disability Units (0.3%). This suggests that, in the surveyed region, the specific neurodevelopmental problems of children with ASD aged 7 to 9 years are recognized by health and educational authorities and that, consequently, most of these children are enrolled in the school system that best fits their special needs. Schools with Autism Units provide specialized support that aims to improve the quality of life of children with ASD, maximizing their autonomy as well as their social interaction and communication skills.

A strength of our study was the high participation rate (88%) of the contacted schools. Participation rate was lower for schools with Multiple Disability Units (76%), when compared to schools with Autism Units (88%) and schools without special needs units (91%). However, as most children diagnosed with ASD hailed from schools with Autism Units, this likely had little impact on prevalence estimation. A possible limitation of our study lies in the use of the TNF as the teachers’ structured tool to identify the children from their classes for further assessment. The TNF showed a reasonable Positive Predictive Value of 65%, meaning that teachers nominated a relatively high percentage of children for whom an ASD diagnosis was not confirmed after clinical assessment (30/85, 35%). This suggests that teachers were very inclusive in the nomination of potential ASD cases, and that omission of positive diagnoses was low. However, we do not know how many of the children that were nominated but not clinically evaluated because of parental refusal might have ASD. In fact, we had an overall low consent rate from parents (96/195; 49%), barring the clinical assessment of potential positive cases, which constituted a major limitation of our study. Parental adhesion was higher for Schools with Autism Units (63.2%), when compared to schools with Multiple Disability Units (38.1%) and schools without special needs units (26.7%). A possible explanation for the overall low participation is that parents may be overwhelmed with their everyday routines or with caring for a child with neurodevelopmental problems. Teachers also referred that a denial process for the existence of a disability was a factor, especially for younger children or when the dysfunction is not very severe. This is supported by the difficulty that teachers reported in conveying the importance of the study to the parents. Similar limitations were reported by other ASDEU studies, carried out in the Tuscany region (28) and Basque Country (27). A higher parental adhesion rate from schools with Autism Units may originate from two reasons: 1) parents from these schools may be more aware of the importance of autism research; 2) children nominated from these schools already have a diagnosis of ASD, so the stage of parental denial has been resolved. Accordingly, it has previously been reported that parents of ASD children have very high participation rates in surveys (25). Higher parental participation could have been achieved through open-sessions in every participating school, establishing a direct contact between the research team and the parents. However, we were unable to do this for such a large sample size and high number of participating schools.

We found a male-to-female ratio of 2.9:1, which is lower than the ratio of 4:1 generally referenced in the literature (35, 36). However, while a male predominance in ASD diagnoses is recognized, some studies suggest the ratio may be closer to 3:1. For instance, in studies that used an active case ascertainment strategy seeking to identify autistic people within a given population, the male-to-female ratio was 3.3:1 (35). Some other prevalence studies have previously obtained ratios similar to ours: 2.5:1 in the general population of 7–12 years old children from South Korea (11) and 2.6:1 and 2.8:1 in registry-based studies from the Stockholm County (Sweden) (37) and Iceland (38), respectively. A sex bias in ASD diagnosis, in which females who meet criteria for ASD are at higher risk of not receiving a diagnosis has also been widely discussed (35, 39). Underlying reasons may be the ability of high-functioning females to camouflage their autistic traits through coping mechanisms to better manage social interactions and a bias of diagnostic instruments toward features typically associated with males (40, 41).

The comparison between the current prevalence report in the Centro region of Portugal and a national epidemiological study carried out in the 1999–2000 school year (30) is a very interesting opportunity to address the underlying causes of the consistently observed increase in ASD prevalence over time (42–45). The study by Oliveira et al. screened 67,795 children aged 6–9 years from mainland Portugal and the Azores archipelago. Of these, 10,585 were from Centro region. Similar to the current study, the strategy adopted involved a school-based screening in which teachers would refer students based on a checklist questionnaire followed by direct clinical assessment by the same clinical team using the same diagnostic instruments. The prevalence estimates for the Centro region obtained by Oliveira et al. (0.125, 95% CI: 0.096–0.15) were approximately four times lower than the present estimates. A possible explanation for the prevalence disparity may lie in differences in the nomination step by teachers. At the time of the study by Oliveira et al., awareness of teachers regarding the specificities of behavior traits in the autism spectrum was much lower. For instance, children with milder forms like Asperger Syndrome were rarely identified in 2000 and may have been missed because they were not recognized by teachers as being in the autism spectrum, and therefore were not nominated. Many of the children nominated by teachers, for whom an ASD diagnosis was not confirmed, met instead criteria for ID. The current greater awareness of ASD likely stems from a broader research on autism that transformed an unclear condition into a better-understood disorder, influencing people’s attitudes toward ASD (46). Overall, the reasons behind the increase in ASD prevalence have been extensively debated and, while a real prevalence increase due to etiological factors, particularly environmental influences, cannot be ruled out (20, 47, 48) it is now accepted that a part of the apparent increase is explained by non-etiological factors. Such factors include the broadening of the spectrum due to evolving diagnostic criteria, and greater public awareness, especially toward ASD in females and younger ages at diagnosis (45). The comparison between the two Portuguese studies supports this notion. The possibility of a direct comparison between studies carried out 17 years apart with similar methodologies highlights the impact on prevalence estimates of diagnostic criteria and disease nosology, as well as training and awareness of education professionals.

Two other published school-based screening studies were performed under the ASDEU project, from the metropolitan area of Pisa (Tuscany region, Italy) (28) and County of Gipuzkoa (Basque Country, Spain) (27), and report prevalence estimates of 1% (95% CI: 0.74–1.34) and 0.59% (95% CI: 0.48–0.73), respectively. It should be noted that the catchment area in the present study spans both urban and rural settings, and has a much larger total population (*N* = 2.2 million) than the metropolitan area of Pisa (*N* = 182,000) or the County of Gipuzkoa (*N* = 715,148) studies. The present study included many more schools (*N* = 1,129 vs 160 in the metropolitan area of Pisa and 182 in the County of Gipuzkoa) and students (*N* = 77,717, vs 10,138 in the metropolitan area of Pisa and 14,734 in the County of Gipuzkoa). Additionally, the aim of the first step of the metropolitan area of Pisa study was to identify, using data from the Local Unit of the Ministry of Education, the total number of children (aged 7–9 years) with an ASD diagnosis attending schools in Pisa. In contrast, in this study we had the participation of 88% of the schools with Autism Units and 76% of the schools with Multiple Disability Units, and consequently expect that a small number of ASD cases may not have been identified.

The ASDEU project also estimated ASD prevalence using data from three nationwide registries from Denmark, Finland and Iceland, and two regional registries from South-West and South-East France (26). Prevalence estimates were similar in Centro region and South-East France (0.48%; 95% CI: 0.40–0.56), slightly higher in South-West France (0.73%; 95% CI: 0.60–0.87) and Finland (0.77%; 95% CI: 0.70–0.84), higher in Denmark (1.26%; 95% CI: 1.17–1.35) and much higher in Iceland (3.13%; 95% CI: 2.64–3.68). Comparing these results with the prevalence estimated for the Portugal Centro region is difficult, as different study designs can have an impact in prevalence estimation (23, 36). Registry-based studies have the advantage of avoiding laborious recruitment and screening processes, while obtaining large sample sizes, but do not detect new cases. Age at diagnosis, which was unavailable for the referred study, is another difficulty for comparison of registry with population screening approaches, because prevalence rates have been shown to increase with age (37, 49). In fact, screening school-aged children is more reliable than at earlier stages or later in life (50). A survey performed under the ASDEU study showed that professionals report that 25% of children only receive a diagnosis at 46 months or older (50). Overall, while the prevalence of 0.5% obtained for Centro region in Portugal is within the range of the other published ASDEU prevalence estimates, comparisons between school-based screening studies or with studies based on registries must be done carefully due to factors that might impact final estimates.

Using a harmonized epidemiological approach for prevalence estimation allows for easier comparisons of prevalence estimates among different regions and countries. This was achieved in the ASDEU study to a certain extent, as the so far published prevalence estimates were homogeneous across the regions using the community-based approach. Some other ASDEU countries, namely France, Finland, Denmark and Iceland, have good centralized and up to date health registries, which allowed the identification of ASD cases using a registry-based approach. While registry-based approaches are able to target larger populations, and are less time-consuming, they may be more easily impacted by local educational and health practices, as well as documentation practices and evolving inclusion criteria, which cannot easily be controlled (26). Overall, except for the higher prevalence in Iceland, both study designs yielded comparable prevalence estimates between 0.48% and 1.26%, reinforcing that either design is valid.

In a global context, in spite of a wide variability of ASD prevalence, our results are comparable to many other countries. A recent literature review found ASD prevalence estimations ranging between 0.01% to 4.36% across the world (10). For instance, prevalence estimations in western Pacific regions, including Australia, are in the order of 2.03%, while in the Americas, Europe and Eastern Mediterranean they are 0.82%, 0.63% and 0.86% respectively, and in South-East Asia prevalence is estimated at 0.34%. Large registries and national surveillance systems like those from the Centers of Disease Control and Prevention (CDC) in the United States (51) and the Public Health Agency in Canada (52), tend to report higher estimates for ASD, more comparable to the Nordic countries of Europe. Zeiden et al. (10) suggest that methodological and contextual differences are responsible for this variation, and that there is not a single methodology that is completely robust. These authors discuss several orders of reasons that may explain some of the discrepancies in prevalence estimation worldwide, beyond methodological differences and more related to the community context. These involve the level of awareness, the education and health system capacity and practices, including differential access to services with a diagnosis of ASD, and the evolving clinical definition of autism over time.

Future efforts should be made to extend a similar strategy to the remaining Portugal regions, as the estimation of ASD prevalence per region is fundamental to a fair provision of education and health services adequate to each region demands. In fact, in the study from 1999 to 2000 (30), we identified regional asymmetries regarding the estimated prevalence values, which were higher in the regions of the Azores (0.156%), Centro (0.125%) and Lisboa e Vale do Tejo (0.123%) and lower in the regions of Alentejo (0.07%), Norte (0.06%) and Algarve (0.024%). It is important to understand if such discrepancies remain and, if so, to identify the reasons at multiple levels. For instance, at the social level reasons could be uneven awareness regarding the pathology among regions, leading some teachers to nominate less or more children with ASD. At the biological level, genetic specificities in the Portuguese population, such as different frequencies of mutations relevant for ASD among the regions due to consanguinity, which is a well-studied phenomenon in the Azores (53), as well as environmental influences may be factors that need to be assessed.

Through the ASDEU project, the prevalence of ASD was estimated in specific regions of European countries, similarly to other studies that also define regional catchment areas (54–56). The ASDEU project provides meaningful information for the wider healthcare and education communities, as reporting results from multiple individual regions is fundamental to attain a broader perspective of the field in Europe. While local estimates are essential for policymakers and healthcare professionals to develop effective interventions, diagnostic approaches, and support systems, altogether they allow the identification of the best practices and the development of guidelines that can be implemented more broadly, promoting consistency and quality in the care and support of individuals with ASD across Europe. A deeper examination of differing prevalence estimates obtained in different ASDEU regions will be necessary to provide information regarding potential risk and protective factors that may differ across countries and regions. Such factors may include socioeconomic factors, access to healthcare, cultural practices, as well as eventual genetic background differences across the studied populations or varying environmental exposures.

As ASD is a lifelong disorder, epidemiological studies aimed at adolescent and adult populations (57) are also relevant. ASD prevalence rates in adults show considerable variation, ranging from 1.1% in a community-based survey (58) to much higher values in psychiatric inpatients (2.4–9.9%) (59). Also, while in most ASD cases symptoms are evident in early childhood, for some subjects, particularly in those with a less severe phenotype, clear manifestation of symptoms might only occur later in life (36, 60). Thus, as the number of ASD cases does not plateau in older populations, having accurate prevalence estimates is vital to ensure that the lifespan needs of these individuals are met.

Finally, it would be important to repeat the prevalence study every 10 years, to assess trends in autism prevalence and provide evidence for programming of adequate health, educational and other resources for individuals with ASD.

## Data availability statement

The raw data supporting the conclusions of this article will be made available by the authors, without undue reservation.

## Ethics statement

The studies involving humans were approved by the Comissão de Ética para a Saúde do Instituto Nacional de Saúde Doutor Ricardo Jorge. The studies were conducted in accordance with the local legislation and institutional requirements. Written informed consent for participation in this study was provided by the participants’ legal guardians/next of kin.

## Author contributions

CR, MP, AV, and GO performed the conceptualization. CR and CC performed the data collection. CR, JS, and CC performed the data curation. CR, CC, AO, FD, and GO performed the clinical evaluations. CR, JS, AN, and AV performed the formal analysis. AN, GO, and AV performed the supervision. CR and JS performed the writing – initial drafting. CR, JS, CC, AO, FD, AN, GO, MP, and AV performed the writing – review and editing. All authors contributed to the article and approved the submitted version.

## Funding

The Autism Spectrum Disorders in the European Union project – ASDEU has been funded by the DG-SANTÉ, European Commission (grant number SANCO/2014/C2/035). This research was supported by national funds from FCT, Fundação para a Ciência e a Tecnologia, I.P. (UIDB/04046/2020 grant to BioISI).

## Conflict of interest

The authors declare that the research was conducted in the absence of any commercial or financial relationships that could be construed as a potential conflict of interest.

## Publisher’s note

All claims expressed in this article are solely those of the authors and do not necessarily represent those of their affiliated organizations, or those of the publisher, the editors and the reviewers. Any product that may be evaluated in this article, or claim that may be made by its manufacturer, is not guaranteed or endorsed by the publisher.

## Footnotes

^1^https://www.pordata.pt/en/What+are+NUTS

^2^2018 census, https://www.pordata.pt/Portugal.
